# Relationship Between Sarcopenia and Electrocardiographic Abnormalities in Older People: The Bushehr Elderly Health Program

**DOI:** 10.3389/fmed.2021.656181

**Published:** 2021-05-07

**Authors:** Ramin Heshmat, Gita Shafiee, Afshin Ostovar, Noushin Fahimfar, Saba Maleki Birjandi, Mohammad Jabbari, Farshad Sharifi, Iraj Nabipour, Bagher Larijani

**Affiliations:** ^1^Chronic Diseases Research Center, Endocrinology and Metabolism Population Sciences Institute, Tehran University of Medical Sciences, Tehran, Iran; ^2^Osteoporosis Research Center, Endocrinology and Metabolism Clinical Sciences Institute, Tehran University of Medical Sciences, Tehran, Iran; ^3^Elderly Health Research Center, Endocrinology and Metabolism Population Sciences Institute, Tehran University of Medical Sciences, Tehran, Iran; ^4^The Persian Gulf Marine Biotechnology Research Center, Bushehr University of Medical Sciences, Bushehr, Iran; ^5^Endocrinology and Metabolism Research Center, Endocrinology and Metabolism Clinical Sciences Institute, Tehran University of Medical Sciences, Tehran, Iran

**Keywords:** sarcopenia, ECG abnormalities, older people, risk, components of sarcopenia

## Abstract

**Background:** Sarcopenia is characterized by low skeletal muscle mass and function, which is associated with cardiovascular risk factors and may even be related to adverse cardiovascular events and mortality. This study aimed to evaluate whether sarcopenia is related to electrocardiographic (ECG) abnormalities in a large sample of older adults.

**Methods:** We performed a cross-sectional study based on the data collected during the Bushehr Elderly Health (BEH) cohort study. Body composition was measured by dual X-ray absorptiometry (DXA) and muscle strength was measured using a digital dynamometer for each hand of every participant. A person who had low muscle strength, as well as low muscle mass was identified as having sarcopenia. The subjects were classified into three groups according to the Minnesota Code (MC) as major, minor ECG abnormalities and participants with no abnormalities ECG.

**Results:** Of the 2,426 participants, 354 (14.6%) had major ECG abnormalities and 193 (8%) had minor ECG abnormalities. Sarcopenia was associated with an increased risk of major ECG abnormality in all models. After adjustment for confounders of CHD in full model, the OR for major ECG abnormality was 1.47 (95% CI 1.11–1.95) in those with sarcopenia. Low muscle strength and low muscle performance were both with an increased risk of major ECG abnormality in all models. Sarcopenia and low muscle strength increased 28% and 62% risk of any ECG abnormality in the full models [sarcopenia: 1.28(1.01–1.63), low muscle strength: 1.62(1.30–2.03)], respectively.

**Conclusions:** This study showed that sarcopenia and its components are associated with ECG abnormalities in Iranian older people. Although some older adults have higher cardiovascular risk factors, these data showed that further factors such as sarcopenia may be identified as a particular risk factor for future cardiovascular events. Therefore, sarcopenia could be added to the screening of the older population to reduce the risk of cardiovascular events.

## Introduction

Aging is related to changes in body composition, including decreases in muscle mass and bone mass and an increase in adipose tissue, which can lead to cardiovascular diseases and metabolic disorders (1).

Sarcopenia, an age-related muscle disorder characterized by a decline in muscle mass and function (2), is a major risk factor of falling, disability and death in old adults. Also, sarcopenia is associated with cardiometabolic risk factors such as glucose intolerance and metabolic syndrome and other diseases such as, cardiovascular diseases (CVD) and respiratory diseases (3).

Among all CVDs, Coronary Heart disease (CHD) is one of the most common and important causes of mortality, morbidity, and disability in older people (4).

Some studies determined cardiometabolic risk factors and comorbidities that associated with CHD (5, 6). The non-modifiable factors such age, sex, race, family history, and modifiable risk factors include obesity, physical inactivity, smoking, hyperlipidemia, hypertension, and diabetes are traditional cardiovascular disease risk factors (5). Also, electrocardiograms (ECG) abnormalities can predict of all- cause, CHD mortality independent of other cardiomeytabolic risk factors (7–11).

Few studies have shown that the association between low muscle mass or sarcopenia and various heart disease in older people (12–14). Some mechanisms including changes in anabolic androgenic hormones, insulin resistance, protein intake, physical activity and muscle structure can be common between CVDs and sarcopenia and can also impact the outcomes of patients with these diseases (15–17).

Although the prevalence and association of ECG abnormalities with chronic diseases such as diabetes and metabolic syndrome and its components have been studied (18, 19), this association has not been investigated in persons with sarcopenia. If ECG abnormalities are common and have associated with sarcopenia, these might help to identify of sarcopenic persons who are at especially high risk for the CVD events. Therefore, to identify subjects with increased risk of CVDs and study on the association between age-related diseases such as sarcopenia with CHD can improve public health problems in elderly.

Given the high prevalence of CHD and also sarcopenia in the Iranian elderly, in the present study, we investigated the association of sarcopenia and its components with ECG abnormalities in older people.

## Materials and Methods

### The Study Population

This cross-sectional study was based on the Bushehr Elderly Health (BEH) program. The BEH study was a prospective population-based cohort study performed on a sample of older people ≥60 years in the urban population of Bushehr city, in the south of Iran. Overall 3,000 persons were recruited using a multistage, stratified cluster sampling method. BEH study aimed to assess non-communicable diseases in older people. The methodology and protocol of the BEH program were previously described elsewhere (20, 21).

The Research Ethics Committee of Bushehr and Tehran University of Medical Sciences approved the protocol of the BEH program (ID:IR.TUMS.EMRI.REC.1394.0036) and written informed consent was signed by all participants.

### Data Collection

A comprehensive questionnaire including sociodemographic characteristics, medical history, smoking and lifestyle data was completed for each person through a personal interview. Anthropometric measurements were carried out using standard protocols. Height and weight were measured with a fixed stadiometer and a digital scale respectively. The body mass index (BMI) was calculated as weight in kg divided by the square of height in meters.

The measurement of Blood pressure (BP) was performed using a standardized mercury sphygmomanometeron the right arm after 15 min of rest in the seated position.

Standard 12-lead electrocardiograms (ECGs) were recorded at baseline in the resting supine position according to the standard procedures. Two qualified physicians coded the ECGs in parallel according to the Minnesota codes using a measuring loop, specially manufactured by the University of Minnesota (22). Any discordant results were resolved by a third qualified physician who was a cardiologist. The physical activity was evaluated by a validated questionnaire based on metabolic equivalent task (MET) (23, 24). A 24-h dietary recall was used for dietary assessment.

A trained operator measured the body composition using dual x-ray absorptiometry (DXA, Discovery WI, HologicInc, USA). The skeletal muscle mass index (SMI) was defined as the sum of the muscle masses of the four limbs as appendicular skeletal muscle mass divided by squared height. Maximum handgrip strength was measured in both hands by a digital grip strength dynamometer, 3 times, and the highest value was used as muscle strength. Walking speed over 4.57 m was used for estimating physical performance.

The biochemical parameters were measured by laboratory testing in a fasting condition, according to standard protocols. Serum lipid profiles and fasting plasma glucose (FPG) were measured by an enzymatic colorimetric technique using a commercial kit (Pars Azmun, Karaj, Iran).

### Definition of Variables

According to the European Working Group on Sarcopenia in Older People 2 (EWGSOP- 2), sarcopenia was defined as low muscle strength plus reduced skeletal muscle mass (3). Also, the EWGSOP and Asia Working Group for Sarcopenia (AWGS) (25) recommend the use of reference data of the same population to determine cut- off points for muscle mass. Therefore, we used reference data from a normative Iranian population that was available for detecting sarcopenia for our study. Based on these data, the cut-off values for low skeletal muscle mass index (SMI) were 7.0 kg/m^2^ and <5.4 kg/m^2^ among men and women, respectively (26). The low muscle strength was handgrip strength <26 kg for men and <18 kg for women; while the cut-off value for low physical performance was a usual walking speed <0.8 m/s for both genders (25). According to these cut-off points, we identified sarcopenic persons (27). Current smoking was defined as smoking cigarettes or water pipes, at the time of study.

Diabetes mellitus was defined as fasting plasma glucose (FPG) ≥ 126 mg/dl or HbA1C ≥ 6.5 or current use of pharmacological medication. Hypertension (HTN) was defined as systolic blood pressure 140 mmHg and/or diastolic blood pressure 90 mmHg or current use of antihypertensive medication. Subjects with total cholesterol ≥200 mg/dl were named as hypercholesterolemia. High Fat Mass was outlined as total body percent fat >30 for men and >40 for women (28).

The Minnesota coding system and Whitehall criteria was used to classify ECG findings as having a major or minor abnormality (22, 29).

Criteria for major ECG abnormalities were any of the following: Q-QS wave abnormalities (MC 1-1 to 1-2-8); left ventricular hypertrophy (MC 3-1); Wolff–Parkinson–White syndrome (MC 6- 4-1 or 6-4-2); complete bundle branch block or intraventricular block (MC 7-1-1, 7-2-1, 7-4, or 7-8); atrial fibrillation or atrial flutter (MC 8-3); or major ST-T changes (MC 4-1, 4-2, 5-1, and 5-2).

Criteria for minor ECG abnormalities were minor ST-T changes (MC 4-3, 4-4, 5-3, and 5-4).

Participants with only minor ECG abnormalities were classified as having “minor abnormalities,” and participants with major ECG abnormalities with or without coexisting minor ECG abnormalities were classified as having “major ECG abnormalities.” Participants with both major and minor abnormalities were classified as having major abnormalities. Participants without minor or major ECG abnormalities were classified as having no abnormalities and their ECG was considered normal (22, 30).

### Statistical Analysis

Normal distribution of continuous variables was assessed using the Shapiro-Wilks test and visual inspection of the group histograms. Continuous data that followed a normal distribution was described with means ± standard deviation (SD). Categorical variables are expressed as percentages. Comparisons between ECG abnormality categories were made using the *t*-test for continuous variables and the chi-squares tests for percentages. We used the best subset method with the Akaike Information Criterion (AIC), to select the final model from all possible subsets. The multinominal regression analyses were used to investigate the associations of sarcopenia and its components with ECG abnormality category. Results were presented as odds ratios and 95% confidence intervals. Data were analyzed using the Stata 14 software (StataCorp. 2015. Stata Statistical Software: Release 14. College Station, TX: StataCorp LP) and *P* ≤ 0.05 was considered as statistically significant in all tests.

## Results

General characteristics of the study group are shown in Table 1. Among 2,426 participants, 193 (8%) had minor ECG abnormalities and 354 (14.6%) had major ECG abnormalities. Subjects in the group with minor or major ECG abnormalities were older and had fewer years of education and also the prevalence of HTN and obesity was higher among these subjects (*P* < 0.05). Participants with minor or major ECG abnormalities walked more slowly and had lower means of SMI and muscle strength (*P* < 0.001).

**Table 1:** General characteristics of the study population according to ECG status.

	**ECG Category**			
	**Normal (No ECG abnormality) (*n* = 1,879)**	**Minor ECG abnormality (*n* = 193)**	**Major ECG abnormality (*n* = 354)**	***P*-value**
Sex(Men),%	939(50.0)	50(25.9)	177(50.0)	0.104
Age, Years	69.14 ± 6.32	69.95 ± 6.69	70.12 ± 6.60	0.012
BMI, Kg/m^2^	27.50 ± 4.90	27.92 ± 5.22	27.37 ± 4.73	0.436
Education, Years	5.37 ± 5.05	4.58 ± 4.87	4.64 ± 4.92	0.008
Physical activity, %	439(23.4)	46(23.8)	70(19.8)	0.186
Current Smoking, %	387(20.6)	39(20.2)	78(22.1)	0.584
Diabetes, %	587(31.8)	61(32.3)	129(36.9)	0.177
Hypercholesterolemia, %	628(33.4)	67(34.7)	99(28.0)	0.110
Hypertension, %	1349(71.9)	144(74.6)	277(78.5)	0.033
High Fat mass, %	1,263(68.5)	145(77.1)	253(73.3)	0.016
Sarcopenia,%	414(22.3)	42(22.2)	106(30.5)	0.004
Low muscle performance, %	867(46.4)	104(54.7)	202(57.9)	<0.001
Low muscle Strength, %	754(40.2)	102(52.8)	191(54.3)	<0.001
Low SMI, %	907(49.2)	82(43.6)	184(53.5)	0.089
SMI, kg/m^2^	6.28 ± 0.98	5.95 ± 0.89	6.18 ± 0.99	<0.001
Muscle strength, Kg	24.32 ± 9.66	20.22 ± 8.30	22.21 ± 9.09	<0.001
Muscle Performance, m/s	0.86 ± 0.31	0.80 ± 0.31	0.78 ± 0.29	<0.001

*Data are n (%) or mean ± standard deviation*.

*CHD, coronary heart disease; BMI, body mass index; SMI, skeletal muscle index*.

**Figure 1:**
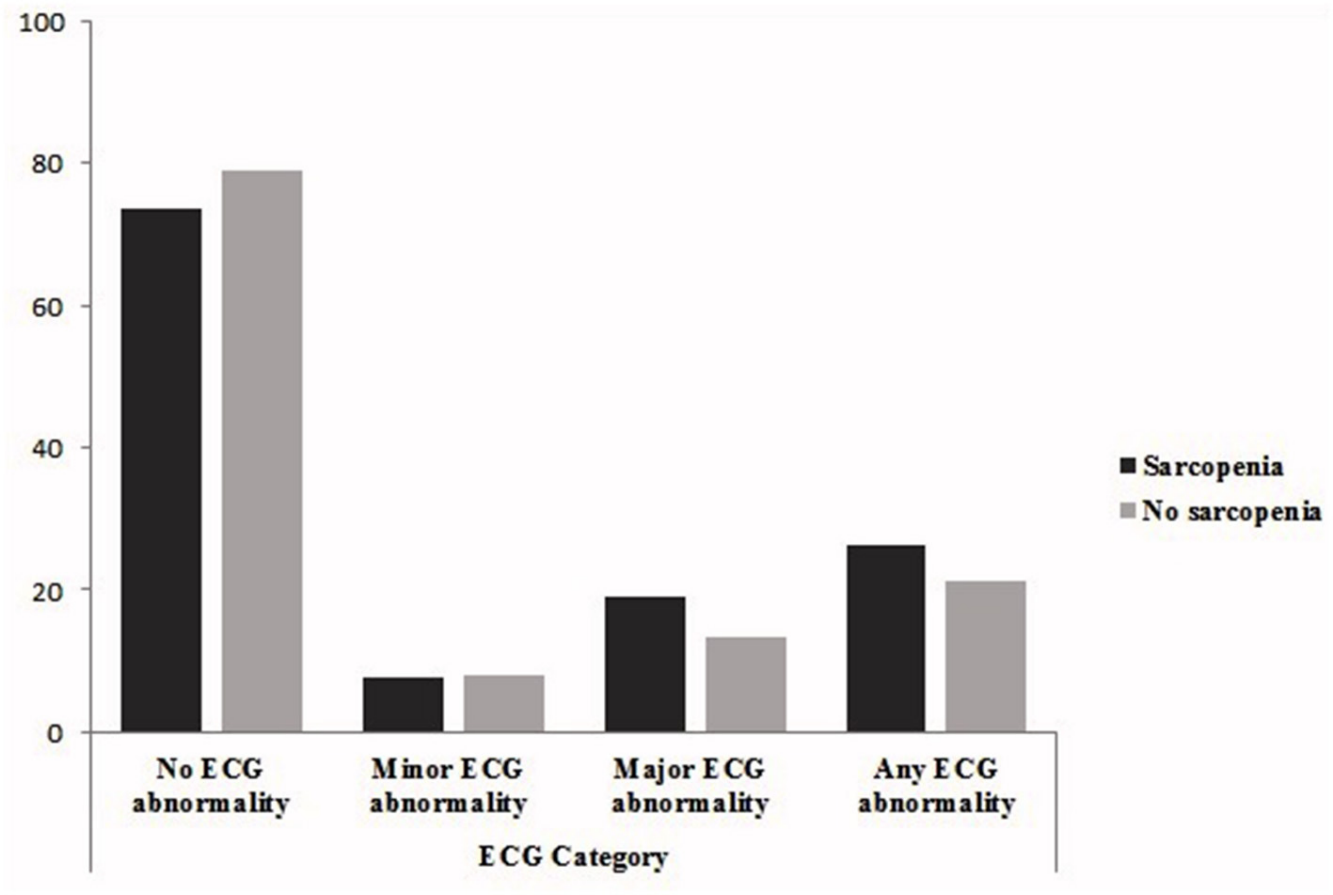
Prevalence rates of sarcopenia according to ECG abnormality category.

Figure 1 shows that the prevalence major or any ECG abnormalities was higher in sarcopenic patients (major ECG abnormalities: 18.9 vs. 13.2%, any ECG abnormalities: 26.3 vs. 21.2%).

Table 2 presents the results of the multinomial logistic regression models to define the association sarcopenia and its components with minor and major ECG abnormalities. Sarcopenia was associated with an increased risk of major ECG abnormality in all models. After adjustment for confounders of CHD in full model, the OR for major ECG abnormality was 1.47 (95% CI 1.11–1.95) in those with sarcopenia. The associations between sarcopenia and minor ECG abnormality were not statistically significant in all models.

**Table 2:** Association of sarcopenia and muscle components with minor and major ECG abnormalities.

	**No ECG abnormality**	**Minor ECG abnormality**	**Major ECG abnormality**
**Sarcopenia**			
Crude model	1.00	0.99(0.69–1.43)	**1.53(1.19**–**1.97)**
Model 1	1.00	0.90(0.61–1.31)	**1.43(1.09**–**1.87)**
Model 2	1.00	0.96(0.65–1.43)	**1.47(1.11**–**1.95)**
**SMI**			
Crude model	1.00	**0.70(0.60**–**0.82)**	0.90(0.80–1.01)
Model 1	1.00	0.90(0.75–1.08)	0.88(0.77–1.02)
Model 2	1.00	0.87(0.72–1.06)	**0.82(0.71**–**0.96)**
**Muscle strength**			
Crude Model	1.00	**0.95(0.93**–**0.97)**	**0.98(0.96**–**0.99)**
Model 1	1.00	0.98(0.96–1.01)	**0.96(0.94**–**0.98)**
Model 2	1.00	0.98(0.95–1.00)	**0.96(0.94**–**0.97)**
**Muscle performance**			
Crude Model	1.00	**0.52(0.31**–**0.85)**	**0.41(0.28**–**0.60)**
Model 1	1.00	1.06(0.59–1.90)	**0.38(0.25**–**0.60)**
Model 2	1.00	1.09(0.59–2.02)	**0.45(0.29**–**0.72)**
**Low SMI**			
Crude model	1.00	0.80(0.59–1.08)	1.18(0.94–1.49)
Model 1	1.00	0.98(0.71–1.34)	1.13(0.89–1.45)
Model 2	1.00	1.01(0.72–1.41)	1.25(0.97–1.61)
**Low muscle strength**			
Crude model	1.00	**1.66(1.24**–**2.24)**	**1.76(1.40**–**2.22)**
Model 1	1.00	1.20(0.86–1.67)	**1.78(1.38**–**2.31)**
Model 2	1.00	1.33(0.94–1.88)	**1.82(1.39**–**2.37)**
**Low muscle performance**			
Crude model	1.00	**1.39(1.03**–**1.88)**	**1.58(1.26**–**1.20)**
Model 1	1.00	0.93(0.66–1.29)	**1.60(1.23**–**2.07)**
Model 2	1.00	0.90(0.64–1.28)	**1.39(1.07**–**1.82)**

*Data are OR and 95% confidence interval. SMI, skeletal muscle index*.

*Model 1; adjusted for age, sex*.

*Model 2; adjusted for age, sex, smoking, physical activity, Education, hypertension, Diabetes, high fat mass, hyperchoesterolemia*.

*The bold values show that the ORs are significant*.

Low muscle strength and low muscle performance were both with an increased risk of major ECG abnormality in all models. Also, the results show that muscle strength and muscle performance decrease risk of major ECG abnormality in all models.

Table 3 demonstrates the association of sarcopenia and muscle components with any ECG abnormality. Sarcopenia and low muscle strength increased 28 and 62% risk of any ECG abnormality in the full models [sarcopenia: 1.28(1.01–1.63), low muscle strength: 1.62(1.30–2.03)], respectively. The relationship between low muscle performance and any ECG abnormality was significant only in crude and age, sex adjusted model. The associations between low SMI and any ECG abnormality were not statistically significant in all models.

**Table 3:** Association of sarcopenia and muscle components with any ECG abnormalities.

	**No ECG abnormality**	**Any ECG abnormality**
**Sarcopenia**		
Crude model	1.00	**1.33(1.07**–**1.65)**
Model 1	1.00	1.22(0.97–1.54)
Model 2	1.00	**1.28(1.01**–**1.63)**
**SMI**		
Crude model	1.00	**0.82(0.75**–**0.91)**
Model 1	1.00	0.89(0.79–1.00)
Model 2	1.00	**0.84(0.74–0.95)**
**Muscle strength**		
Crude Model	1.00	**0.97(0.96**–**0.98)**
Model 1	1.00	**0.97(0.95**–**0.98)**
Model 2	1.00	**0.96(0.95**–**0.98)**
**Muscle performance**		
Crude model	1.00	**0.44(0.32**–**0.61)**
Model 1	1.00	**0.54(0.37**–**0.79)**
Model 2	1.00	**0.61(0.42**–**0.91)**
**Low SMI**		
Crude Model	1.00	1.03(0.85–1.25)
Model 1	1.00	1.08(0.88–1.32)
Model 2	1.00	1.16(0.94–1.43)
**Low muscle strength**		
Crude Model	1.00	**1.73(1.43**–**2.09)**
Model 1	1.00	**1.55(1.251.92)**
Model 2	1.00	**1.62(1.30**–**2.03)**
**Low muscle performance**		
Crude model	1.00	**1.51(1.25**–**1.84)**
Model 1	1.00	**1.32(1.06**–**1.63)**
Model 2	1.00	1.19(0.95–1.49)

*Data are OR and 95% Confidence Interval. SMI; Skeletal Muscle Index*.

*Model 1; adjusted for age, sex*.

*Model 2; adjusted for age, sex, smoking, physical activity, education, hypertension, diabetes, high fat mass, hyperchoesterolemia*.

*The bold values show that the ORs are significant*.

Also, we observed statistically significant associations of SMI, muscle strength and muscle performance with any ECG abnormality in all models especially in full models.

## Discussion

This cross-sectional study aimed to assay the association of sarcopenia and components of muscle with major and minor ECG abnormalities. The results of the present study demonstrated sarcopenia to be independently and strongly associated with major ECG abnormalities in older people. Furthermore, participants with low muscle strength or low muscle performance had higher risk of major ECG abnormality than those with normal ECG.

Studies explained that skeletal muscle, as the most tissue in the human body, is involved in some metabolic functions including energy expenditure, protein metabolism and insulin sensitivity (31, 32). Also, these muscles have endocrine actions with releasing some myokines and play in regulation inflammation and immune function. Therefore, decline of muscle mass and muscle function, as named sarcopenia can lead to several age-related metabolic disorders and some diseases such as CHD, hypertension and heart failure (33–35).

CHD is a major cause of death and disability in the world. Understanding the risk factors and comorbidities is essential for prevention, early diagnosis, reduction of mortality and to evaluate management effectiveness (4). Among comorbidities, sarcopenia with common mechanisms can associate with CHD. Previous reports have shown that the associations of sarcopenia and its parameters with carotid atherosclerosis, myocardial infarction (MI), chronic heart failure and other heart diseases (14, 33). A recent study found that sarcopenia was associated with MI and atrial fibrillation. However, in the mention study, sarcopenia was defined based on the skeletal muscle mass and muscle strength did not assay in their population (14). In another study, researchers showed that sarcopenia was as prognostic predictor in older people with acute MI (12).

ECG is an available, low cost and useful tool for risk prediction of asymptomatic subjects with CHD, especially in older people given their higher prevalence of CVD events (36, 37). In some studies, CHD was defined as a symptom of angina pectoris based on the Rose Angina Questionnaire, a positive history of CHD, or a positive ECG for CHD. There are also studies that use only one definition of CHD separately. Therefore, CHD could be defined based on ECG abnormalities (29, 38, 39).

In this study, we revealed the relationship between sarcopenia and major ECG abnormalities. The model analysis indicated that the risk of major ECG abnormality was increased in sarcopenic participants (OR = 1.47, 95% CI: 1.11–1.95) even after adjustment for several risk factors. Also, among components of sarcopenia, we found the independent association between muscle strength and muscle performance with ECG abnormalities. Considerable evidence supports muscle strength, an important component of sarcopenia, has an independent role in the prevention of cardiovascular events and mortality (40–42). A systematic review of 23 selected publications showed that muscle strength was inversely and independently associated with all cause and cardiovascular mortality. Furthermore, a strong and inverse association of muscle strength with mortality had been confirmed in patients with chronic diseases such as CHD, cancer, and peripheral artery disease (43).

Our findings also supported that low muscle mass alone may not completely reveal muscle performance and that muscle strength should be considered an important parameter in defining sarcopenia. This issue has also been considered by the EWGSOP and in the recent definition of sarcopenia, the importance of muscle strength over muscle mass has been emphasized (3). Furthermore, the present study demonstrated the important of muscle performance as a risk factor for CHD. It implied that the muscle function of the lower extremities may be important in cardiovascular outcomes. Consistent with our findings, recent studies revealed gait speed was associated with an increased HRs for death and cardiovascular mortality (44, 45).

Both muscle strength and muscle performance are used to assess the function of skeletal muscle and these tests are simple and rapid and can be performed in older people. On the other hand, due to the relevance of these parameters with health outcomes, these are useful for predicting CVD and all- cause mortality. This may, at least in part, explain our observations that muscle strength and muscle performance are independently and significantly associated with health outcomes regardless of muscle mass (45, 46).

The principal mechanism for the relationships between sarcopenia and its components, especially muscle strength or muscle performance, with CHD has not been clearly explored. Muscle mitochondrial dysfunction, which is involved in the pathogenesis of sarcopenia, may also play a role to develop of cardiovascular disease by increasing oxidative stress production and damage of vascular endothelium (47). In addition, insulin resistance, chronic inflammation, and abnormalities in anabolic hormones such as low testosterone levels are other common mechanisms involve in sarcopenia especially low muscle function and atherosclerosis diseases (48–50). Physical activity and activities of daily living may be reduced after developing CVDs and also muscle atrophy occurs by reducing blood flow in skeletal muscle and infiltration of adipocytes into muscle fibers (50). The above mechanisms suggest that sarcopenia and its parameters associate with CVDs and prevention and treatment of sarcopenia would decrease the risk of atherosclerosis and death in older people.

In this study, some limitations should be considered. The cross-sectional design limited the possibilities of determining the causal inferences and further longitudinal designs are needed to clarify any causal relationship. However, ECG is an easy screening test for CHD, but not the gold standard method such as coronary angiography. Therefore, this issue might be impacted on our results. Despite these limitations, the strength of this study is that our findings were based on a great sample size from a population-based study provided data on musculoskeletal disorders such as sarcopenia among older people in Iran. Also, skeletal muscle mass was directly measured with DXA as a gold standard for diagnosing low muscle mass.

## Conclusions

This study highlighted that sarcopenia was associated with ECG abnormalities independent of the well-known cardiovascular risk factors in Iranian older people. Among parameters of sarcopenia, muscle strength and muscle performance were the most important factors to associate with ECG abnormalities.

## Data Availability Statement

The raw data supporting the conclusions of this article will be made available by the authors, without undue reservation.

## Ethics Statement

The studies involving human participants were reviewed and approved by Bushehr University of Medical Sciences. The patients/participants provided their written informed consent to participate in this study.

## Author Contributions

All authors listed have made a substantial, direct and intellectual contribution to the work, and approved it for publication.

## Conflict of Interest

The authors declare that the research was conducted in the absence of any commercial or financial relationships that could be construed as a potential conflict of interest.
